# Interferon and interferon-induced cytokines as markers of impending clinical progression in ANA^+^ individuals without a systemic autoimmune rheumatic disease diagnosis

**DOI:** 10.1186/s13075-023-02997-w

**Published:** 2023-02-10

**Authors:** Sonya T. Kim, Carolina Muñoz-Grajales, Shannon E. Dunn, Raphael Schneider, Sindhu R. Johnson, Zahi Touma, Zareen Ahmad, Dennisse Bonilla, Eshetu G. Atenafu, Linda T. Hiraki, Arthur Bookman, Joan Wither

**Affiliations:** 1 Schroeder Arthritis Institute, Krembil Research Institute, University Health Network, 60 Leonard Avenue, Toronto, ON M5T 0S8 Canada; 2 Department of Immunology, Faculty of Medicine, University of Toronto, Toronto, ON Canada; 3 Keenan Research Centre for Biomedical Science, St. Michael’s Hospital, Toronto, ON Canada; 4 Division of Neurology, St. Michael’s Hospital Unity Health, Toronto, ON Canada; 5 Department of Medicine, Faculty of Medicine, University of Toronto, Toronto, ON Canada; 6 Toronto Scleroderma Program, Division of Rheumatology, Toronto Western and Mount Sinai Hospitals, Toronto, ON Canada; 7 University of Toronto Lupus Clinic, Centre for Prognosis Studies in Rheumatic Diseases, Schroeder Arthritis Institute, University Health Network, Toronto, ON Canada; 8 Biostatistics Department, Princess Margaret Cancer Center, University Health Network, Toronto, Canada; 9 Division of Rheumatology, The Hospital for Sick Children, and Department of Paediatrics, University of Toronto, Toronto, ON Canada; 10 Division of Rheumatology, Schroeder Arthritis Institute, University Health Network, Toronto, ON Canada

**Keywords:** Systemic autoimmune rheumatic diseases, Pre-clinical, Interferon, Cytokines

## Abstract

**Background:**

Elevated levels of interferons (IFNs) are a characteristic feature of systemic autoimmune rheumatic diseases (SARDs) and may be useful in predicting impending symptomatic progression in anti-nuclear antibody-positive (ANA^+^) individuals lacking a SARD diagnosis. Typically, these are measured by their effect on gene expression in the blood, which has limited their utility in clinical settings. Here, we assessed whether the measurement of serum IFN-α or selected IFN-induced cytokines accurately mirrors IFN-induced gene expression in ANA^+^ individuals and investigated their utility as biomarkers of clinical progression.

**Methods:**

A total of 280 subjects were studied, including 50 ANA^−^ healthy controls, 160 ANA^+^ individuals without a SARD diagnosis (96 asymptomatic, 64 with undifferentiated connective tissue disease), and 70 SARD patients. IFN-induced gene expression was measured by nanoString and cytokine levels by ELISA or Simoa. ANA^+^ individuals lacking a SARD diagnosis who had the new onset of SARD criteria over the subsequent 2 years were defined as progressors.

**Results:**

Measurement of IFN-α levels by high-sensitivity ELISA or Simoa correlated much better with IFN-induced gene expression than measurement of CXCL-10 or Galectin-9 levels. Despite this, high CXCL-10 and Galectin-9 levels were better predictors of subsequent progression in ANA^+^ individuals than measures of IFN-α or IFN-induced gene expression with the optimal combination of predictive cytokines (CXCL-10 and IFN-α as measured by ELISA), resulting in a specificity and positive predictive value of 100%.

**Conclusion:**

Easily performed ELISA assays for CXCL-10 and IFN-α can be used to predict ANA^+^ individuals at high risk of imminent symptomatic progression.

**Supplementary Information:**

The online version contains supplementary material available at 10.1186/s13075-023-02997-w.

## Background

The systemic autoimmune rheumatic diseases (SARDs), including systemic lupus erythematosus (SLE), Sjögren’s syndrome (SS), and systemic sclerosis (SSc), have overlapping clinical features and are thought to have a similar etiopathogenesis. One of the characteristic immune abnormalities in these conditions is the presence of anti-nuclear antibodies (ANAs), which can be produced many years in advance of clinical disease onset. For example, ANAs are seen up to 9 or 18 years prior to the diagnosis of SLE or SS, respectively [1–4]. While these findings suggest that ANAs might be useful for early detection of SARD, ANAs are also prevalent within the general population (~20% of women) [5], with only a minority of these individuals (<10%) going on to develop SARD in their lifetime based upon the prevalence of these conditions. Since the clinical onset of SARD is associated with significant morbidity, and occasionally irreversible tissue damage, biomarkers that accurately predict disease development could improve patient outcomes by allowing for the initiation of treatment prior to this damage.

Elevated levels of interferons (IFNs) are a characteristic feature of SARD [6–10]. These elevations are also seen in a subset of ANA^+^ individuals that lack a diagnosis of SARD [5, 11–13] and in some studies have been shown to be associated with an enhanced risk of progression to SARD. In a study of ANA^+^ individuals, most of whom had at least one baseline symptom of SARD, elevated levels of IFN-induced gene expression in the peripheral blood were predictive of progression to SLE or SS over the subsequent year [13]. Studies in our laboratory have shown that the levels of IFN-induced gene expression are higher in asymptomatic ANA^+^ individuals that developed new SARD criteria on follow-up within the next 2 years than those who did not [14]. While these studies suggest that elevated IFN levels may act as a predictive biomarker for SARD progression, the techniques required for the measurement of IFN have limited their clinical utility. Recently, serum levels of Galectin-9 and CXCL-10 were found to be elevated in patients with SLE or the antiphospholipid syndrome and were shown to strongly correlate with IFN-induced gene expression in the peripheral blood [15], suggesting that measurement of these cytokines can be used as surrogate markers for IFN-induced gene expression. In addition, technical advances in the measurement of IFN-α, one of the major IFNs that promotes the elevated IFN-induced gene expression in SARD, have enabled the measurement of this cytokine in ranges that were previously undetectable. In this study, we assessed the correlation between these new measures of IFN and those used in our previous studies in a well-characterized cohort of ANA^+^ individuals with and without a SARD diagnosis and evaluated their ability to predict symptom progression in a subset of ANA^+^ individuals lacking a SARD diagnosis followed longitudinally.

## Methods

### Subjects and data collection

From July 2013 to May 2019, individuals referred for rheumatologic assessment due to a recently discovered positive ANA test were serially recruited and consented, at the Toronto Western and Mount Sinai Hospitals. All demographic and clinical information was recorded on a standardized data retrieval form. The ANA was re-measured by immunofluorescence (IF), at the University Health Network laboratory, and individuals with an ANA ≥ 1:160 were stratified into 3 groups: (1) individuals lacking ANA-associated SARD criteria (the 1997 ACR criteria for SLE [16], the 2013 ACR-EULAR criteria for SSc [17], or the 2016 ACR-EULAR criteria for SS [18]), termed ANA^+^NS (*n* = 96); (2) individuals with ≥ 1 SARD criteria, but insufficient criteria for a SARD diagnosis (*n* = 64, undifferentiated connective tissue disease, UCTD); or (3) early (within 2 years of diagnosis) untreated (except anti-malarials) SARD patients (*n* = 70). Healthy controls (*n* = 50, ANA^−^HC) were confirmed to be ANA negative (≤ 1:40) by immunofluorescence (IF) and using the Bioplex® 2200 ANA Screening System (Bio-Rad Laboratories, Hercules, CA), with those with a positive test being reclassified into the ANA^+^NS group. For ANA^+^NS and UCTD individuals recruited after July 2015, yearly follow-up, with an extensive clinical evaluation, measurement of autoantibodies by Bioplex®, and banking of blood for research, was offered. Individuals who developed new ANA-associated SARD criteria during the subsequent 2 years were considered clinical progressors. The study was approved by the Research Ethics Boards of both recruiting hospitals.

### Measurement of autoantibodies

ANAs were quantified by indirect IF using the Kallestad® HEp-2 kit (Bio-Rad Laboratories) in the University Health Network laboratory. The Bioplex® 2200 ANA Screening System was used to measure the serum levels of 11 specific autoantibodies (anti-dsDNA, anti-chromatin, anti-Ro, anti-La, anti-Sm, anti-SmRNP, anti-RNP, anti-Jo-1, anti-Scl-70, anti-centromere, and anti-ribosomal P), with a positive test being determined using the company’s cut-offs (antibody index ≥ 1). The levels of anti-Ro52 antibodies were measured using a custom autoantigen array, as previously published [19]. Briefly, Ro52 (Diarect, Surmodics IVD, Eden Prarie, MN) was spotted in duplicate onto two-pad FAST nitrocellulose coated slides (GVS, Sanford, ME) using a VersArray Chipwriter Pro Microarrayer (Virtek, Canada). After drying, the slides were blocked overnight at 4°C in PBS with 5% fetal calf sera and 0.1% Tween-20 and then incubated with serum samples diluted 1:100 in blocking buffer for 1 h at 4°C. After washing, slides were probed with Cy3-labeled goat anti-human IgG (Jackson ImmunoResearch, West Grove, PA; diluted 1:2000 in blocking buffer) for 45 min at 4°C, washed again, and dried by centrifugation. Every batch had negative (PBS) and positive (human serum with known reactivity to ribosomal P0; Immunovision, Springdale, AZ) controls. Fluorescent intensities were quantified using an Axon 4200A microarray scanner (Molecular Devices, Sunnyvale, CA) and net fluorescent intensities (NFI) were calculated from raw data by subtracting local background. Samples were performed in duplicate and the replicates were combined to generate a mean NFI. To control for batch effects, the positive control was used to generate a normalization factor.

### Measurement of IFN-induced gene expression

Gene expression in total RNA isolated from whole peripheral blood archived in Tempus tubes (Applied Biosystems, Waltham, MA, USA) was quantified using a custom array (NanoString Technologies, Seattle, WA, USA), as previously described [5]. Log_2_ normalized expression levels of 5 interferon (IFN)-induced genes (*EPSTI1*, *IFI44L*, *LY6E*, *OAS3*, *RSAD2*) were summed to generate a composite IFN5 score.

### Cytokine measurement

Archived samples (never thawed, stored at −80 °C) were used for all cytokine measurements. Levels of IFN-α were determined in the serum using a high-sensitivity VeriKine human IFN-α ELISA Kit (PBL Assay Science, Piscataway, NJ, USA), which detects all 12 human IFN-α subtypes (dynamic range 1.95–125 pg/mL), and were also quantified using a Simoa HD-1 instrument with the Simoa® IFN-α Advantage Kit HD-1/HD-X (Quanterix Corp., Billerica, MA, USA), which has a linear range from 0 to 60 pg/mL and an LLOQ = 0.016 pg/mL. Levels of Galectin-9 and CXCL-10 (IP-10) were measured in diluted serum (1/5 dilution) using DuoSet ELISA kits (R&D Systems, Minneapolis, MN, USA), with dynamic ranges of 93.8–6000 pg/mL and 31.2–2000 pg/mL, respectively. The levels of IFN-γ in neat serum were determined using a Quantikine High Sensitivity ELISA kit (R&D Systems), following the manufacturer’s instructions (dynamic range 0.5–30 pg/mL). Except for some of the Simoa assays, all measurements were performed in duplicate.

### Statistical analysis

Statistically significant differences between groups were determined by the Mann-Whitney *U* test (for 2 groups) and the Kruskal-Wallis test followed by Dunn’s post-test for multiple comparisons (≥ 3 groups). The association between measurements of different cytokines was determined using Spearman’s rank correlation coefficient. A multivariable regression model was used to assess the impact of sex and ethnicity on the levels of cytokines between different groups. To assess the relative ability of elevated levels of CXCL-10, Galectin-9, and/or IFN-α alone or in combination to predict progression, a logistic regression model was used with the cut-offs determined by Youden’s index, calculated by comparing progressors and non-progressors. All *p* values were 2-sided and a *p* < 0.05 was considered to be statistically significant. Statistical analyses were performed using GraphPad Prism version 8.3.1 (GraphPad Software, San Diego, CA, USA) and version 9.4 of the SAS system for Windows (Copyright © 2002–2012 SAS Institute Inc., Cary, NC, USA).

## Results

Cytokine levels were measured on a total of 280 subjects; 151 overlapped with our previous study in which we measured IFN-induced gene expression (Table 1) [5]. Most of the study subjects were female and of Caucasian ethnicity, with a greater proportion of males and a lower proportion of Caucasians in the ANA^−^HC, as compared to the other ANA^+^ participant subsets. Fifty-five of the ANA^+^ individuals lacking a SARD diagnosis that were followed longitudinally had completed 2 years of follow-up at the time of the study, 13 of which developed new SARD criteria during this period. The new clinical criteria that developed and resulting diagnoses for the progressors are outlined in Additional file 1: Suppl. Table 1. For both progressors and non-progressors, the majority of individuals were female and Caucasian. Progressors had significantly more autoantibody specificities, as measured by Bioplex, than non-progressors and were more likely to be of South Asian ancestry. The proportion of UCTD patients within the subset of ANA^+^ individuals lacking a SARD diagnosis that demonstrated progression (37.7%) was not significantly different from that in non-progressors (53.8%, *p* = 0.33), with similar clinical features being present at baseline in both groups (Additional file 1: Suppl. Table 2) and similar proportions of individuals receiving antimalarial therapy (23% in progressors and 16.7% in non-progressors, *p* = 0.68).

**Table 1:** Study participant characteristics

				SARD				Followed ≥ 2 years	
	ANA^**−**^HC	ANA^**+**^NS	UCTD	SARD (total)	SSc	SS	SLE	Non-progressors	Clinical progressors
	*N*=50	*N*=96	*N*=64	*N*=70	*N*=22	*N*=30	*N*=16	*N*= 42	*N*=13
**Sex: female *n*(%)**	35 (70)	**91 (94.8)**	**59 (92.2)**	**65 (92.9)**	20 (90.9)	27 (90)	**16 (100)**	40 (95.2)	11 (84.6)
**Age: mean ± SD**	31.5 ± 11.5	**43.2 ± 13.8**	**44.0 ± 15.0**	**49.4 ± 14.6**^**a**^	**54.8 ± 13.5**^**a,b**^	**51.7 ± 12.7**^**a**^	38.4 ± 14.9	43.0 ± 14.8	46.8 ± 11.4
**Ethnicity:*n*(%)**									
Caucasian	21 (42)	**58 (60.4)**	**47 (73.4)**	**49 (70)**	**16 (72.7)**	**23 (76.7)**	9 (56.3)	29 (69.0)	7 (53.8)
Afro-Caribbean	1 (2)	**13 (13.5)**	4 (6.3)	1 (1.4)^a^	0 (0)	0 (0)^a^	1 (6.3)	3 (7.1)	0 (0)
East Asian	2 (4)	8 (8.3)	5 (7.8)	5 (7.1)	1 (4.5)	3 (10)	1 (6.3)	1 (2.4)	1 (7.7)
South Asian	14 (28)	**13 (13.5)**	**6 (9.4)**	**8 (11.4)**	2 (9.1)	3 (10)	2 (12.5)	5 (11.9)	5 (38.5)^c^
Hispanic	9 (18)	**2 (2.1)**	**2 (3.1)**	5 (7.1)	3 (13.6)^a^	1 (3.3)	1 (6.3)	2 (4.8)	0 (0)
Other	0 (0)	2 (2.1)	0 (0)	1 (1.4)	0 (0)	0 (0)	1 (6.3)	2 (4.8)	0 (0)
No data	3 (6)	0 (0)	0 (0)	1 (1.4)	0 (0)	0 (0)	1 (6.3)	0 (0)	0 (0)
**Anti-Ro Ab**^**+**^**mother**^**d**^**:*n*(%)**	0 (0)	8 (8.3)	2 (3.1)	0 (0)	0 (0)	0 (0)	0 (0)	2 (4.8)	1 (7.7)
**ANA IF titer: median**	0	**1/640**	**1/640**	**>1/640**	**>1/640**	**1/640**	**1/640**	1/640	1/640
**Number of specific Abs: mean ± SD**	0	**0.76 ± 1.12**	**1.06 ± 1.14**^**a**^	**1.88 ± 1.35**^**a,b**^	**1.14 ± 0.85**	**2.00 ± 0.64**^**a,b**^	**2.56 ± 2.19**^**a,b**^	0.74 ± 0.83	1.54 ± 1.51^c^
**Specific Abs:*n*(%)**									
dsDNA	0 (0)	8 (8.3)	3 (4.7)	**9 (12.9)**	2 (9.1)	**3 (10)**	**4 (25)**^**b**^	4 (9.5)	0 (0)
Ro	0 (0)	**28 (29.2)**	**20 (31.3)**	**41 (58.6)**^**a,b**^	**3 (13.6)**	**30 (100)**^**a,b**^	**8 (50)**	13 (31.0)	7 (53.8)
La	0 (0)	**12 (12.5)**	4 (6.3)	**22 (31.4)**^**a,b**^	0 (0)	**21 (70)**^**a,b**^	1 (6.3)	4 (9.5)	4 (30.8)
Sm	0 (0)	3 (3.1)	3 (4.7)	**6 (8.6)**	0 (0)	0 (0)	**5 (31.3)**^**a,b**^	0 (0)	2 (15.4)
SmRNP	0 (0)	4 (4.2)	**8 (12.5)**	**9 (12.9)**	1 (4.5)	0 (0)	**7 (43.8)**^**a,b**^	2 (4.8)	2 (15.4)
RNP	0 (0)	**9 (9.4)**	**12 (18.8)**	**11 (15.7)**	1 (4.5)	2 (6.7)	**7 (43.8)**^**a,b**^	2 (4.8)	3 (23.1)
Scl70	0 (0)	1 (1)	2 (3.1)	**9 (12.9)**^**a**^	**5 (22.7)**^**a,b**^	2 (6.7)	2 (12.5)	2 (4.8)	0 (0)
Jo-1	0 (0)	0 (0)	0 (0)	0 (0)	0 (0)	0 (0)	0 (0)	0 (0)	0 (0)
Centromere	0 (0)	1 (1)	**7 (10.9)**^**a**^	**16 (22.9)**^**a**^	**13 (59.1)**^**a,b**^	1 (3.3)	1 (6.3)	1 (2.4)	0 (0)
Chromatin	0 (0)	7 (7.3)	**6 (9.4)**	**8 (11.4)**	0 (0)	1 (3.3)	**6 (37.5)**^**a,b**^	3 (7.1)	2 (15.4)
Ribosomal P	0 (0)	0 (0)	0 (0)	0 (0)	0 (0)	0 (0)	0 (0)	0 (0)	0 (0)
**IFN5 score: median (IQR)**	48.6 (47.1–50.5)	**53.1 (48.9–63.0)**	**53.1 (48.4–61.5)**	**64.6 (53.4–68.5)**^**a,b**^	**54.6 (49.7–60.1)**	**67.4 (61.9–68.8)**^**a,b**^	**65.5 (55.0–67.8)**^**a,b**^	52.9 (48.7–62.0)	56.2 (49.2–64.8)

*Abbreviations*: *ANA* anti-nuclear antibody, *ANA*^*−*^*HC* ANA-negative healthy control, *ANA*^*+*^*NS* asymptomatic ANA-positive individual, *UCTD* undifferentiated connective tissue disease, *SARD* systemic autoimmune rheumatic disease, *SSc* systemic sclerosis, *SS* Sjogren’s disease, *SLE* systemic lupus erythematosus, *N* number, *SD* standard deviation, *Abs* antibodies, *IQR* interquartile range, *NA* not applicable

Significant differences (*p* < 0.05) from ANA^−^HC are bolded

^a^Significantly different from ANA^+^HC (*p* < 0.05)

^b^Significantly different from UCTD (*p* < 0.05)

^c^Significantly different from non-progressors (*p* < 0.05)

^d^Anti-Ro Ab-positive mother of a child with congenital heart block or neonatal lupus without a SARD diagnosis at study recruitment

### IFN-induced gene expression and several of the cytokine measures were elevated in patients with a SARD diagnosis

**Fig. 1:**
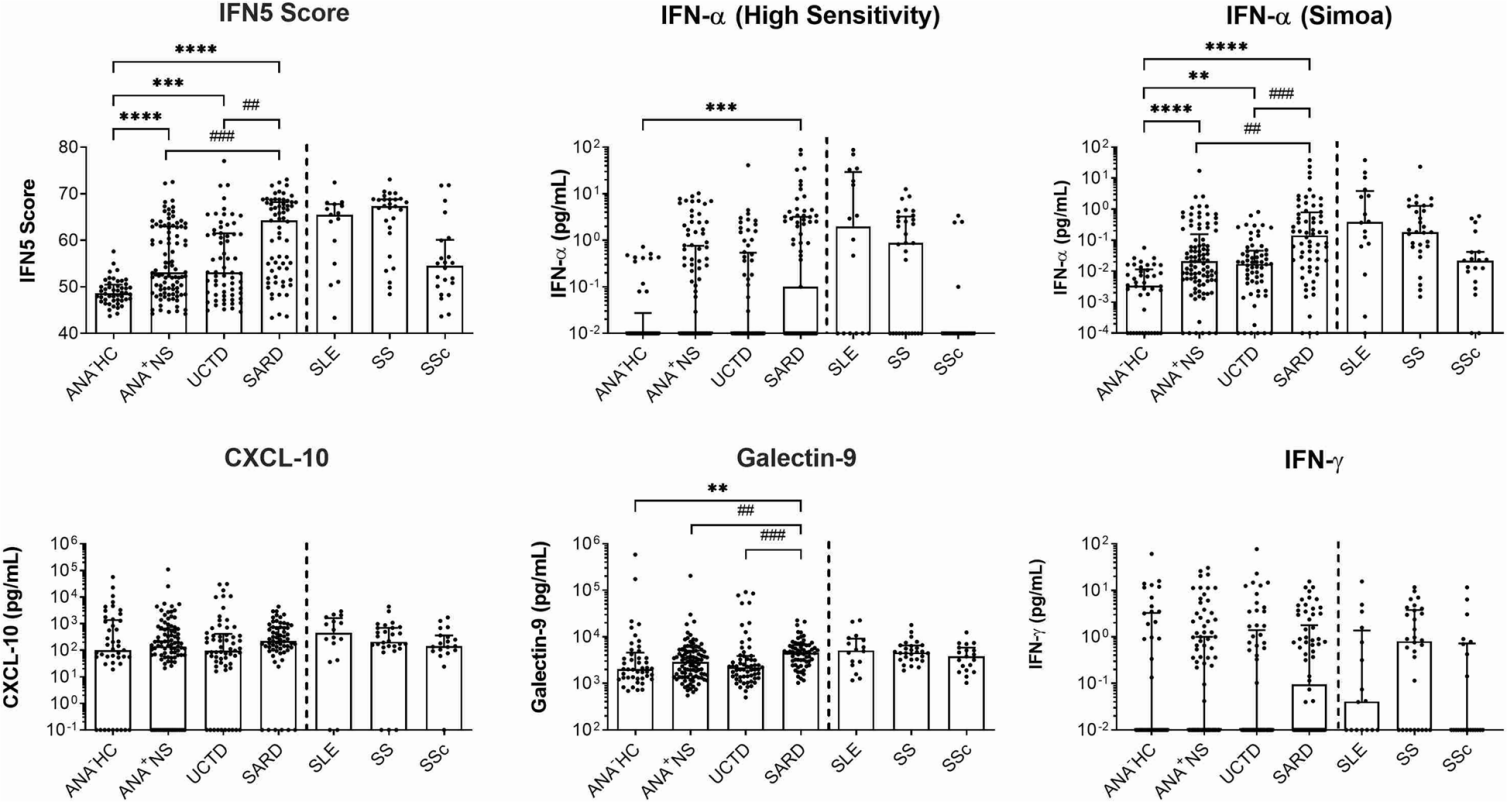
Cytokine levels in the ANA^+^ participant subsets. Scatterplots showing the results for the IFN5 score and the cytokines, IFN-α measured by high-sensitivity ELISA, IFN-α measured by Simoa, CXCL-10, Galectin-9, and IFN-γ (all shown using logarithmic scales). From left to right are shown results for healthy controls (ANA^−^HC), asymptomatic ANA^+^ individuals (ANA^+^NS), undifferentiated connective tissue disease (UCTD) patients, and systemic autoimmune rheumatic disease (SARD) patients. Results to the right of the dashed line are those for the individual SARD conditions, SLE, SS, and SSc. Each circle represents a single subject, with the bars indicating the median for the subjects and error bars denoting the interquartile range. Significant differences between ANA^−^HC and the ANA^+^ groups are indicated with asterisks, *p < 0.05, **p < 0.01, ***p < 0.001, and ****p < 0.0001, and those between SARD and the other ANA^+^ subsets indicated with cross hatches, using the same scale

Figure 1 shows the levels for the various assays used to assess IFN or its effects in all study subjects. As previously shown by ourselves and others, peripheral blood IFN-induced gene expression, as determined by the IFN5 score, was significantly elevated in SARD patients and in ANA^+^ individuals lacking a SARD diagnosis, although not to the same extent as that seen in SARD. IFN-α, as measured by a high-sensitivity ELISA, was also significantly elevated in SARD patients. However, for a considerable number of study participants, the IFN-α levels were below the lower limit of detection of this ELISA. To better capture differences in this lower range, we measured serum IFN-α by Simoa. As expected, this improved the ability to detect IFN-α in more subjects, permitting the identification of elevated levels of IFN-α not only in SARD patients, but also in both subsets of ANA^+^ individuals lacking a SARD diagnosis, relative to ANA^−^HC. In contrast to these more direct measures of type I IFN, the levels of CXCL-10 did not differ from ANA^−^HC in any of the ANA^+^ groups and significantly elevated levels of Galectin-9 were only seen for SARD patients. Similar findings were also obtained for IFN-γ, which was not elevated in any of the ANA^+^ groups.

Given the large differences in age between some of the groups, we performed a multivariable analysis incorporating age as a predictor to determine the impact it had on the results, and for cytokines that were significantly impacted by age, we did an age-adjusted analysis of the statistical differences between groups. The only cytokine measurements that varied significantly with age were IFN-α measured by ELISA (*p*=0.002) and Simoa (*p*=0.021), where an age-adjusted analysis led only to a loss of significance between ANA^−^HC and ANA^+^NS or UCTD for Simoa, but no other comparisons between groups.

We also assessed whether the duration of storage at −80° C affected the levels of cytokines detected by performing a linear regression analysis and found no association between the age of the sample and cytokine levels.

### IFN-α is a good surrogate marker of the IFN signature, whereas CXCL-10 and Galectin-9 reflect both type I IFN and IFN-γ elevations

To further explore the interrelationship between the various surrogate markers of IFN-induced gene expression and the IFN5 score, as well as each other, we examined their correlation in all study subjects (Table 2). There was a moderate to strong correlation between the IFN5 score and IFN-α measured by ELISA or Simoa, which was strongest for Simoa. In contrast, there was only a weak correlation between the IFN5 score and serum levels of CXCL-10, Galectin-9, and IFN-γ.

**Table 2:** Correlations between cytokines for all subjects

Cytokine	With cytokine	Correlation	95% CI		***p*** value
IFN5	CXCL-10	0.12378	0.001679	0.241790	**0.0466**
IFN5	Galectin-9	0.29788	0.182062	0.404577	**<0.0001**
IFN5	IFN-α by ELISA	0.54516	0.454252	0.623581	**<0.0001**
IFN5	IFN-α by Simoa	0.70824	0.640511	0.763888	**<0.0001**
IFN5	IFN-γ	0.12436	0.002743	0.241896	**0.0447**
CXCL-10	Galectin-9	0.74356	0.683753	0.792397	**<0.0001**
CXCL-10	IFN-α by ELISA	0.18992	0.070689	0.303147	**0.0019**
CXCL-10	IFN-α by Simoa	0.15592	0.033657	0.273010	**0.0125**
CXCL-10	IFN-γ	0.28330	0.167120	0.390802	**<0.0001**
Galectin-9	IFN-α by ELISA	0.30807	0.194242	0.412745	**<0.0001**
Galectin-9	IFN-α by Simoa	0.23755	0.117934	0.349549	**0.0001**
Galectin-9	IFN-γ	0.27885	0.162421	0.386702	**<0.0001**
IFN-α by ELISA	IFN-α by Simoa	0.59586	0.510414	0.668379	**<0.0001**
IFN-α by ELISA	IFN-γ	0.09315	−0.027377	0.210669	0.1290
IFN-α by Simoa	IFN-γ	0.04034	−0.082748	0.162064	0.5209

Spearman’s correlation coefficient for the association between the indicated cytokines, together with the 95% confidence interval (CI). Significant *p* values are bolded

Given the discordance between our results and those previously reported in the literature for predominantly SLE patients [15], we questioned whether the correlation between these cytokines and the IFN5 score varied based on the diagnosis. In ANA^+^NS, there was no correlation between either CXCL-10 or Galectin-9 and the IFN5 score, whereas there was a weak correlation for Galectin-9 with the IFN5 score in UCTD and SARD patients (*ρ* = 0.29 and 0.25, respectively, both *p* ≤ 0.05). In contrast, there was a moderate to strong correlation between IFN-α, regardless of how it was measured, and the IFN5 score for all 3 ANA^+^ groups (*ρ* = 0.52–0.71, all *p* < 0.0001).

Previous reports have suggested that CXCL-10 and Galectin-9 are predominantly driven by IFN-γ [20, 21]. When all subjects were included, there was a weak correlation between these two cytokines and IFN-γ, which approximated that seen for the IFN5 score and IFN-α, with no marked differences observed between ANA^+^ individuals with and without symptoms. These findings raise the possibility that the production of these cytokines in ANA^+^ individuals is not solely induced by IFN-γ. Nevertheless, the levels of CXCL-10 and Galectin-9 strongly correlated with each other (Table 2), suggesting that they may arise from similar pathogenic mechanisms.

### Sex and ethnicity affect cytokine levels

Given the difference in the proportion of males between ANA^−^HC and the ANA^+^ subject groups, and previous work by ourselves and others suggesting that ethnicity may affect cytokine levels, we questioned whether these demographic factors could have an impact on cytokine levels. To address this question, a multivariable analysis was performed incorporating sex and ethnicity as covariates. This analysis demonstrated that sex was a contributing factor to the variation in levels of Galectin-9 (*p* = 0.003) and IFN5 score (*p* = 0.035), whereas ethnicity affected the levels of IFN-α measured by ELISA (*p* < 0.0002).

The differences in cytokine levels between males and females and between Caucasian and non-Caucasian subjects for each subject group are shown in Additional file 1: Suppl. Figs. 1 and 2, respectively. Differences between male and female subjects were mostly seen in the ANA^−^HC group, with elevated levels of Galectin-9 and other IFN-γ-associated cytokines being significantly higher in males than females. In contrast, the differences between Caucasian and non-Caucasian subjects were predominantly restricted to the ANA^+^ groups, with not only IFN-α, as measured by ELISA, but also other measures of IFN-α showing variably significant increases in non-Caucasians in these groups. This finding is consistent with previous studies by ourselves and others showing elevated levels of measures of type I IFN in non-Caucasian, as compared to Caucasian, ANA^+^ individuals [5, 22, 23].

### Serum levels of IFN-induced cytokines can be used to predict disease progression in ANA^+^ individuals

**Fig. 2:**
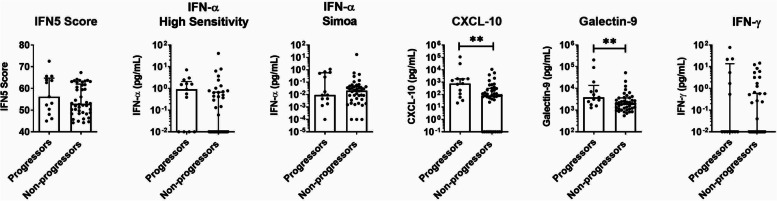
Cytokine levels in longitudinally followed ANA^+^ subjects lacking a SARD diagnosis stratified for the presence or absence of clinical progression over the subsequent 2 years. Levels of the various cytokines in clinical progressors (*n* = 13) and non-progressors (*n* = 42). Scatterplots showing the results for the IFN5 score and the cytokines, IFN-α measured by high-sensitivity ELISA, IFN-α measured by Simoa, CXCL-10, Galectin-9, and IFN-γ (all shown using logarithmic scales). Each circle represents a single subject, with the bars indicating the median for the subjects and error bars denoting the interquartile range. Significant differences between progressors and non-progressors are indicated by asterisks, ** *p* < 0.01

To examine the ability of the different cytokine measures to predict progression in ANA^+^ subjects lacking a SARD diagnosis, the levels of each measure were compared between clinical symptom progressors (*n*=13) and non-progressors (*n*=42), who had completed 2 years of follow-up (Fig. 2). The mean levels of Galectin-9 and CXCL-10 were significantly elevated in progressors compared to non-progressors (*p* = 0.0074 and *p =* 0.0052, respectively), and there was a trend to increased levels of IFN-α, as measured by a high-sensitivity ELISA (*p* = 0.059). To determine whether measurement of these cytokines could be used to predict clinical disease progression in ANA^+^ subjects lacking a SARD diagnosis, receiver operating characteristic curves for each cytokine measure were produced and the optimal cut-off for progression determined by calculating Youden’s index, comparing progressors and non-progressors (see Additional file 1: Suppl. Fig. 3).

The greatest discriminative ability, as indicated by the highest area under the curve, was seen for Galectin-9, CXCL-10, and IFN-α measured by high-sensitivity ELISA, with IFN-γ and IFN-α measured by Simoa showing little or no discriminative ability. Using univariate logistic regression, we determined that ANA^+^ individuals with elevated levels of Galectin-9 (*p* = 0.008, OR = 6.34, 95% CI 1.62–24.80), CXCL-10 (*p* = 0.0017, OR = 9.6, 95% CI 2.34–39.42), or IFN-α (*p* = 0.011, OR=5.83, 95% CI 1.50–22.71) are more likely to progress within the next 2 years. To determine whether a combination of these cytokines offered an improved ability over the individual cytokine measures to predict progression, their sensitivity, specificity, positive predictive value (PPV), and negative predictive value (NPV) were contrasted (Table 3). While the individual cytokines tended to have higher sensitivity, in general, the specificity and PPV improved with combinations of cytokines. Notably, the combination of elevated levels of CXCL-10 and IFN-α was strongly predictive of progression, with a specificity and PPV of 100%. Although the area under the curve for the IFN5 score was only slightly less than that for IFN-α measured by high-sensitivity ELISA and using the optimal cut-off for progression had a comparable specificity and PPV to IFN-α (either alone or in combination with other cytokines), it had a significantly reduced sensitivity and NPV (Additional file 1: Suppl. Table 3).

**Table 3:** Comparison of various combinations of cytokines as predictors of clinical progression in ANA^+^ subjects lacking a SARD diagnosis

Cytokine combination	Sensitivity (%)	Specificity (%)	PPV (%)	NPV (%)
Galectin-9 (≥ 2914 pg/mL)	69.23	73.81	45.00	88.57
CXCL-10 (≥ 689 pg/mL)	61.54	85.71	57.14	87.80
IFN-α (≥ 0.91 pg/mL)	53.85	83.33	50.00	85.37
CXCL-10 and Galectin-9	53.85	88.10	58.33	86.05
Galectin-9 and IFN-α	46.15	97.62	85.71	85.42
CXCL-10 and IFN-α	46.15	100.00	100.00	85.71
RO52 (≥ 11.87 NFI)	75.00	71.79	45.00	90.32
RO52 and Galectin-9	58.33	92.31	70.00	87.80
RO52 and CXCL-10	50.00	92.31	66.67	85.71
RO52 and IFN-α	58.33	87.18	58.33	87.18
RO52 and CXCL-10 and Galectin-9	50.00	94.87	75.00	86.05
RO52 and Galectin-9 and IFN-α	50.00	97.44	85.71	86.36
RO52 and CXCL-10 and IFN-α	46.15	100.00	100.00	85.71
RO52 and CXCL-10 and Galectin-9 and IFN-α	46.15	100.00	100.00	85.71

Sensitivity, specificity, positive predictive value (PPV), and negative predictive value (NPV) for clinical progression in the subsequent 2 years. Only those cytokine or antibody measures that individually were significantly associated with progression are included in the table. All IFN-α measurements were done by high-sensitivity ELISA. Optimal cut-offs were determined by Youden’s index, calculated by comparing progressors and non-progressors

We previously examined the ability of 144 autoantibodies, as measured by an autoantigen array, to predict progression and found that only elevated levels of anti-Ro52 antibodies were associated with an increased risk of development of new SARD criteria over the subsequent 2 years (PPV 46%, NPV 89%) [19]. We therefore investigated whether the measurement of this antibody improved the ability of the cytokine measures to predict progression, using our previously established cut-off. The presence of elevated levels of anti-Ro52 antibodies resulted in very modest increases in the specificity and PPV when combined with the individual cytokine measures, but offered a minimal improvement over the combinations of cytokines. Because we previously noted a moderate correlation between the IFN5 score and the presence of anti-Ro52 antibodies [19], we questioned whether elevated levels of anti-Ro52 antibodies offered the same discriminative ability between progressors and non-progressors as IFN-α. Although the presence of elevated anti-Ro52 antibody levels in tandem with elevated levels of CXCL-10 or Galectin-9 improved the sensitivity of detection of progressors, as compared to the presence of elevated levels of the same cytokine with increased IFN-α levels, it resulted in a significant decrease in the specificity and especially the PPV.

None of the progressors or non-progressors had a dense fine speckled pattern on their ANA and there was no difference in the proportion of subjects with any of the remaining ANA patterns between progressors and non-progressors.

## Discussion

IFNs have been shown to play an important pathogenic role in SARD [24–26], and there is some evidence that their measurement may be useful in predicting the ANA^+^ individuals who lack a SARD diagnosis that are at high risk for subsequent SARD development [13, 14]. However, the measurement of IFN levels in these studies was largely performed by assessing IFN-induced gene expression in the peripheral blood or performing IFN activity assays, which are not easily adapted to clinical use. In this study, we assessed whether the measurement of serum IFN-α levels or IFN-induced cytokines could provide comparable information to the measurement of the IFN signature. We show that serum IFN-α levels correlate much better than the measurement of CXCL-10 or Galectin-9 with IFN-induced gene expression, as measured by the IFN5 score. Despite this, measurements of CXCL-10 and Galectin-9 were better at predicting subsequent development of new SARD criteria in ANA^+^ individuals lacking a SARD diagnosis than measures of IFN-α, with the optimal combination of predictive cytokines being CXCL-10 and IFN-α, as measured by high-sensitivity ELISA, resulting in a specificity and PPV of 100%.

Previous work has shown that the IFN-induced genes that are upregulated in SLE fall into 3 groups, a group that is readily induced by IFN-α, a group that requires a stronger stimulus for induction, such as IFN-β, and a group that is proposed to also require IFN-γ for optimal induction [27, 28]. All of the genes in the IFN5 score fall into the first of these groups, and therefore, it is not surprising that they correlate most strongly with the serum levels of IFN-α. We have previously shown that there is a moderate correlation between serum IFN-α, as measured by high-sensitivity ELISA, and IFN-induced gene expression, with serum IFN-α levels only being detectable by this technique when very high levels of IFN-induced gene expression are present [5]. Here, we reproduce these findings in a larger group of ANA^+^ individuals and show that serum IFN-α, as measured by Simoa, demonstrates an even stronger correlation with IFN-induced gene expression in the blood. It is likely that the increased strength of this correlation results from Simoa’s improved ability to detect low levels of serum IFN-α, as the two assays for IFN-α correlate well with each other in the higher range of detection. Despite this stronger correlation, measurement of IFN-α by Simoa did not improve the ability to discriminate between progressors and non-progressors, as the high levels of IFN-α that discriminate between these two groups were easily detectable by high-sensitivity ELISA.

In contrast to the previous study that formed the basis for our current investigation [15], we detected only a poor correlation between CXCL-10 and Galectin-9 serum levels and IFN-induced gene expression. This was not the result of variation in the association between these cytokines and the IFN5 score in the different ANA^+^ disease states, as no difference in the strength of association was seen between ANA^+^NS or SARD, nor was it the result of differences in the types of IFN-induced genes that were used for comparison, since for both studies the genes that were used to detect the IFN signature were from the cluster of genes that is readily induced by IFN-α, with 2 of 5 genes overlapping between the studies. While the origin of the differences remains unclear, this may result from differences in the extent of disease activity of the SLE patients between the two studies, with those previously studied having well-established disease and substantial disease activity, whereas those in our study had very early untreated disease which was generally milder in severity.

Despite the lack of correlation between CXCL-10 and Galectin-9 with the IFN signature, elevated levels of these cytokines appeared to be good markers of impending clinical progression in ANA^+^ individuals lacking a SARD diagnosis. The relationship between these cytokines and imminent progression suggests that the immune mechanisms leading to their production may play an important role in this process. As outlined previously, the production of CXCL-10 and Galectin-9 can be induced by IFN-γ [20, 21], and therefore, it is possible that immune events that trigger IFN-γ production promote the transition to symptomatic disease. The observation that elevations in the levels of IFN-γ are seen prior to disease onset in SLE patients is compatible with this concept [29]. Additional support for this concept comes from a longitudinal study of ANA^+^ individuals, most of whom had UCTD, where an IFN score that contained genes that are thought to require IFN-γ for their induction better predicted imminent clinical progression than a score containing genes that are easily induced by IFN-α [13].

Alternatively, the immune events leading to the production of CXCL-10 and Galectin-9 may be more complex, involving multiple IFNs, rather than just IFN-γ. This is supported by our observation that there was only a weak correlation between the serum levels of CXCL-10 or Galectin-9 and IFN-γ. While it is possible that the serum levels of IFN-γ do not accurately reflect those produced in the tissues, there is some evidence that other IFNs can act coordinately with IFN-α to induce these cytokines. For example, in the skin, keratinocytes produce CXCL-10 in response to IFN-α [30]. SLE patients and some ANA^+^ individuals lacking a SARD diagnosis have increased levels of IFN-κ in their skin [31], which amplifies the IFN-α response [32]. IFN-α is also known to increase STAT1 expression, which could act more generally to amplify IFN-γ signaling in the cells of multiple tissues [33].

It is likely that the elevated levels of CXCL-10 or Galectin-9 in ANA^+^ progressors indicate the presence of sub-clinical tissue inflammation, which can pre-date the onset of clinical symptoms by months. Although, as outlined above, it is possible that CXCL-10 can be elaborated in the skin in the absence of obvious inflammation, elevations of Galectin-9 are not reported to occur in this setting and are typically seen when there is chronic inflammation or infection [34]. Both CXCL10 and Galectin-9 are produced by a variety of cells that variably include lymphocytes, monocytes, macrophages, dendritic cells, fibroblasts, neutrophils, endothelial cells, and epithelial cells (keratinocytes for CXCL-10 and gastrointestinal for Galectin-9) [34, 35]. Importantly, once CXCL-10 is produced, it attracts monocytes, macrophages, T cells, NK cells, and dendritic cells to the site of inflammation that can further augment the inflammatory process, eventually resulting in sufficient inflammation to produce clinical disease [36]. It is possible that the increased specificity and PPV for CXCL-10 as compared to Galectin-9 for progression, either alone or in combination with IFN-α, reflects a more important role for CXCL-10 in disease progression; however, further study is required to address this possibility.

Surprisingly, the levels of Galectin-9, CXCL-10, and IFN-γ were significantly elevated in male, as compared to female, ANA^−^HC. The reason for these elevations in otherwise healthy males is unknown. In general, the data reported in the literature on these cytokines has not stratified the results with regard to sex, so it is not clear whether this is a sex-determined difference or whether it results from a covariate that we have not been able to identify. However, it is unlikely that a recent infection could account for these findings, since healthy controls with a recent viral infection or immunization were excluded from recruitment. Despite the elevations of these cytokines in male healthy controls, elevated levels of CXCL-10 and Galectin-9, alone or in tandem with IFN-α, appeared to be similarly predictive in males and females, as 1 of 2 ANA^+^ males that progressed had elevations of both these cytokines which was not seen in either of the 2 male non-progressors.

Our study has several limitations. First, the follow-up period for our longitudinally followed pre-clinical ANA^+^ subjects is still relatively short, and therefore, the value of these cytokines in predicting progression beyond 2 years is unknown. Second, as we have not measured these cytokines over time, we do not know how far in advance of progression these cytokine changes can be seen. Third, there was a relatively small number of progressors in our study and validation of our findings in a larger cohort of patients is required.

## Conclusions

There is growing interest in identifying ANA^+^ individuals at high risk for the development of SARD; however, biomarkers that are strongly predictive of imminent disease progression and that can easily be performed on a screening basis are lacking. Our study indicates that easily performed ELISA assays can be used for this purpose and that elevated levels of CXCL-10 and IFN-α are strongly predictive of imminent symptomatic progression. If validated, our findings could pave the way to the use of disease-delaying therapies, such as anti-malarials, and therapeutic trials of potentially preventative therapies prior to disease onset in high-risk individuals, which if successful could prevent much of the tissue damage that occurs early in disease.

## Supplementary Information

**Additional file 1: Supplementary Table 1.** Clinical Characteristics of Progressors. **Supplementary Table 2.** Comparison of Baseline Clinical Characteristics in UCTD progressors and non-progressors. **Supplementary Table 3.** The IFN5 score as a predictor of clinical progression in ANA+ subjects lacking a SARD diagnosis, alone or in combination with other cytokines. **Supplementary Figure 1.** Cytokine levels in the ANA+ participant subsets stratified by sex. Scatterplots showing the results for the IFN5 score and the cytokines, IFN-α measured by high sensitivity ELISA, IFN-α measured by Simoa, CXCL-10, Galectin-9, and IFN-γ (all shown using logarithmic scales). From left to right, are shown results for healthy controls (ANA-HC), asymptomatic ANA+ individuals (ANA+NS), undifferentiated connective tissue disease (UCTD) patients, and systemic autoimmune rheumatic disease (SARD) patients (F, female; M, male). Each circle represents a single subject, with the bars indicating the median for the subjects and error bars denoting the interquartile range. Significant differences between males and females are indicated with asterisks, * *p* < 0.05, ** *p* < 0.01, and *** *p* < 0.001. **Supplementary Figure 2.** Cytokine levels in the ANA+ participant subsets stratified by ethnicity. Scatterplots showing the results for the IFN5 score and the cytokines, IFN-α measured by high sensitivity ELISA, IFN-α measured by Simoa, CXCL-10, Galectin-9, and IFN-γ (all shown using logarithmic scales). From left to right, are shown results for healthy controls (ANA-HC), asymptomatic ANA+ individuals (ANA+NS), undifferentiated connective tissue disease (UCTD) patients, and systemic autoimmune rheumatic disease (SARD) patients (C, Caucasian; NC, non-Caucasian). Each circle represents a single subject, with the bars indicating the median for the subjects and error bars denoting the interquartile range. Significant differences between Caucasians and non-Caucasians are indicated with asterisks, * *p* < 0.05, ** *p* < 0.01, and **** *p* < 0.0001. **Supplementary Figure 3.** Receiver operating characteristic curves for prediction of clinical progression in subjects followed for ≥ 2 years using the various cytokine measures. The clinical and serologic characteristics of the clinical progressor (*n*=13) and non-progressor (*n*=42) subjects are shown in Table 1. Results are shown for the IFN5 score and the cytokines, IFN-α measured by high sensitivity ELISA, IFN-α measured by Simoa, CXCL-10, Galectin-9, and IFN-γ. Numbers in brackets following the cytokine labels and the black dot on the curve indicate the calculated value of Youden’s Index, comparing progressors and non-progressors. The area under the curve (AUC) is shown in the bottom right corner.

## Data Availability

The data used to support the findings of this study are available from the corresponding author upon request.

## Abbreviations

SARD: Systemic autoimmune rheumatic disease
SLE: Systemic lupus erythematosus
SS: Sjogren’s syndrome
SSc: Systemic sclerosis
UCTD: Undifferentiated connective tissue disease
ANA: Anti-nuclear antibody
PPV: Positive predictive value
NPV: Negative predictive value
IFN: Interferon
