# Supplementary material for: Interferon and interferon-induced cytokines as markers of impending clinical progression in ANA+ individuals without a systemic autoimmune rheumatic disease diagnosis
Source: Arthritis Res Ther. 2023 Feb 10;25:21. doi: 10.1186/s13075-023-02997-w (PMC9912609; doi:10.1186/s13075-023-02997-w)
Supplement: Supplementary file 1 — Additional file 1: Supplementary Table 1. Clinical Characteristics of Progressors. Supplementary Table 2. Comparison of Baseline Clinical Characteristics in UCTD progressors and non-progressors. Supplementary Table 3. The IFN5 score as a predictor of clinical progression in ANA+ subjects lacking a SARD diagnosis, alone or in combination with other cytokines. Supplementary Figure 1. Cytokine levels in the ANA+ participant subsets stratified by sex. Scatterplots showing the results for the IFN5 score and the cytokines, IFN-α measured by high sensitivity ELISA, IFN-α measured by Simoa, CXCL-10, Galectin-9, and IFN-γ (all shown using logarithmic scales). From left to right, are shown results for healthy controls (ANA-HC), asymptomatic ANA+ individuals (ANA+NS), undifferentiated connective tissue disease (UCTD) patients, and systemic autoimmune rheumatic disease (SARD) patients (F, female; M, male). Each circle represents a single subject, with the bars indicating the median for the subjects and error bars denoting the interquartile range. Significant differences between males and females are indicated with asterisks, * p < 0.05, ** p < 0.01, and *** p < 0.001. Supplementary Figure 2. Cytokine levels in the ANA+ participant subsets stratified by ethnicity. Scatterplots showing the results for the IFN5 score and the cytokines, IFN-α measured by high sensitivity ELISA, IFN-α measured by Simoa, CXCL-10, Galectin-9, and IFN-γ (all shown using logarithmic scales). From left to right, are shown results for healthy controls (ANA-HC), asymptomatic ANA+ individuals (ANA+NS), undifferentiated connective tissue disease (UCTD) patients, and systemic autoimmune rheumatic disease (SARD) patients (C, Caucasian; NC, non-Caucasian). Each circle represents a single subject, with the bars indicating the median for the subjects and error bars denoting the interquartile range. Significant differences between Caucasians and non-Caucasians are indicated with asterisks, * p < 0.05, ** p < 0.01, [file 13075_2023_2997_MOESM1_ESM.docx]

**Supplementary Table 1.** Clinical Characteristics of Progressors

| **ANA^+^NS (6)** | | | |
| --- | --- | --- | --- |
| **Baseline criteria** | **Sex** | **New criteria** | **Final diagnosis** |
| Nil | Female | Arthritis, inflammatory rash,  Raynaud's phenomenon | SLE |
| Nil | Female | Alopecia, photosensitivity,  Raynaud's phenomenon | SLE |
| Nil | Female | Arthritis, Raynaud's phenomenon | UCTD |
| Nil | Female | Unilateral Schirmer ≤ 5 mm/5min | SS |
| Nil | Female | Schirmer ≤ 5 mm/5min | SS |
| Nil | Male | Thrombocytopenia | UCTD |
| **UCTD (7)** | | | |
| **Baseline criteria** | **Sex** | **New criteria** | **Final diagnosis** |
| Raynaud's phenomenon, abnormal nailfold capillaries | Male | Puffy fingers | UCTD |
| Abnormal salivary pool (anti-Ro negative) Peripheral neuropathy | Female | Arthritis | UCTD |
| Raynaud's phenomenon | Female | Nasal ulcers, arthritis | UCTD |
| Raynaud's phenomenon, esophageal dysmotility | Female | Lower tract Intestinal dysmotility, skin tightness | SSc |
| Parotitis | Female | Arthritis, Schirmer ≤ 5 mm/5min | SS |
| Raynaud's phenomenon, abnormal nailfold capillaries, puffy fingers | Female | Fingertip pitting | SSc |
| Photosensitivity | Female | Parotitis | UCTD |

**Supplementary Table 2.** Comparison of Baseline Clinical Characteristics in UCTD progressors and non-progressors

| **Criteria** | **Progressors**  **n (%)** | **Non-progressors**  **n (%)** |
| --- | --- | --- |
| Abnormal nailfold capillaries | 2 (25) | 3 (20) |
| Raynaud's phenomenon | 4 (50) | 5 (33) |
| Interstitial lung disease | 0 (0) | 2 (13) |
| Esophageal dysmotility | 1 (12.5) | 0 (0) |
| Puffy fingers | 1 (12.5) | 1 (6) |
| Saliva flow rate ≤ 0.1 ml/min | 1 (12.5) | 0 (0) |
| Parotitis | 1 (12.5) | 0 (0) |
| Alopecia | 0 (0) | 1 (6) |
| Photosensitivity | 1 (12.5) | 2 (13) |
| Inflammatory rash | 1 (12.5) | 1 (6) |
| Oral ulcers | 0 (0) | 1 (6) |
| Arthritis | 0 (0) | 4 (26.6) |
| Pleuritis/pericarditis | 0 (0) | 2 (13) |
| Neuropathy | 1 (12.5) | 0 (0) |

**Supplementary Table 3.** The IFN5 score as a predictor of clinical progression in ANA^+^ subjects lacking a SARD diagnosis, alone or in combination with other cytokines.

| **Cytokine Combination** | **Sensitivity**  **(%)** | **Specificity**  **(%)** | **PPV**  **(%)** | **NPV**  **(%)** |
| --- | --- | --- | --- | --- |
| IFN5 (≥ 63.64 pg/mL) | 38.46 | 90.48 | 55.46 | 82.61 |
| IFN5 and Galectin-9 | 30.77 | 95.45 | 66.67 | 82.35 |
| CXCL-10 and IFN5 | 30.77 | 95.45 | 66.67 | 82.35 |
| RO52 and IFN5 | 41.67 | 89.74 | 55.56 | 83.33 |
| RO52 and IFN5 and Galectin-9 | 33.33 | 97.44 | 80.00 | 82.61 |
| RO52 and CXCL-10 and IFN5 | 33.33 | 97.44 | 80.00 | 82.61 |
| RO52 and CXCL-10 and IFN5 and Galectin-9 | 33.33 | 100.00 | 100.00 | 82.98 |

Sensitivity, specificity, positive predictive value (PPV), and negative predictive value (NPV) for clinical progression in the subsequent 2 years. Only those cytokine or antibody measures that individually were significantly associated with progression are included in the table. Optimal cut-offs were determined by Youden’s Index, calculated by comparing progressors and non-progressors.


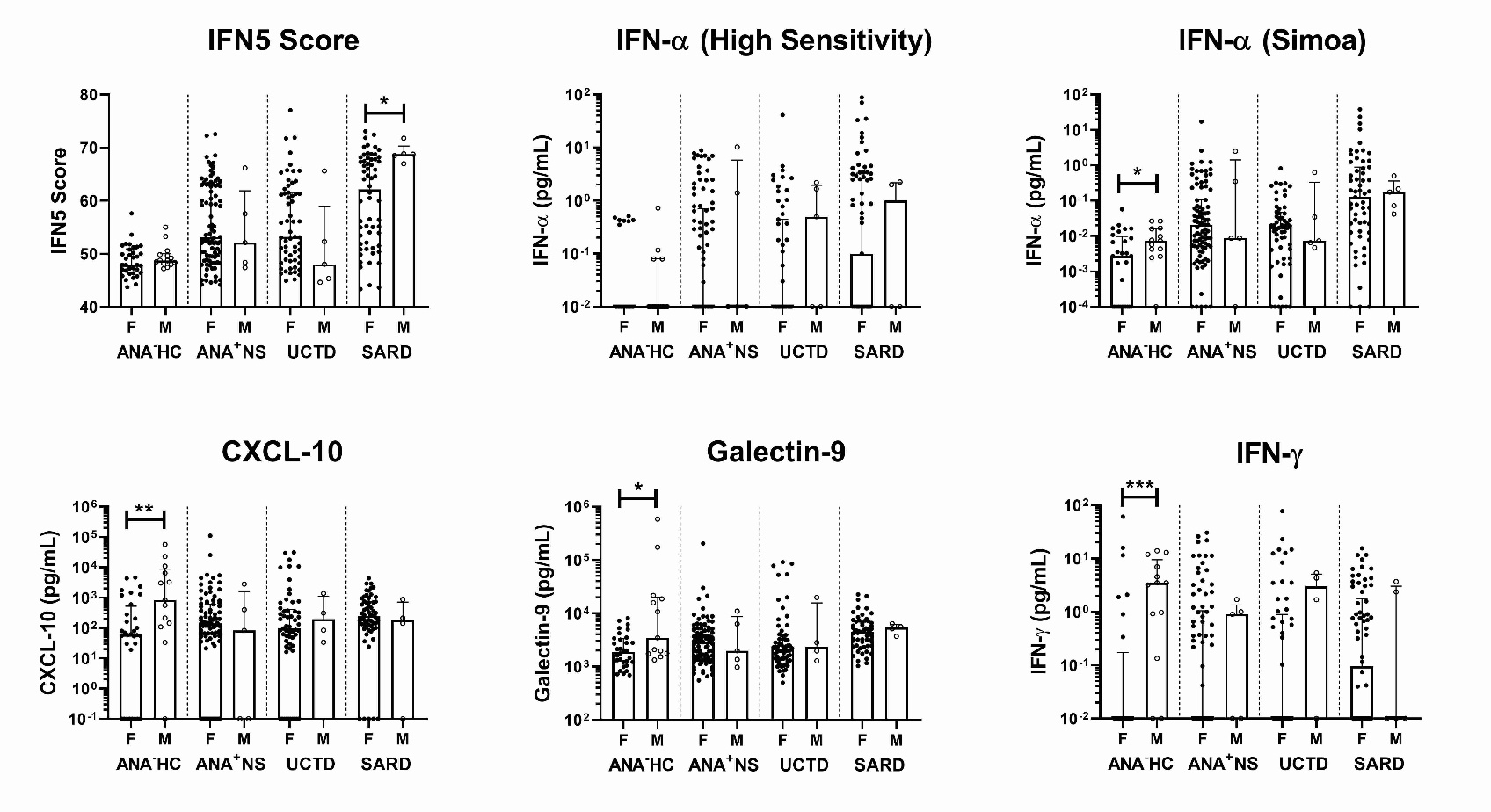


**Supplementary Figure 1.** **Cytokine levels in the ANA^+^ participant subsets stratified by sex.** Scatterplots showing the results for the IFN5 score and the cytokines, IFN-α measured by high sensitivity ELISA, IFN-α measured by Simoa, CXCL-10, Galectin-9, and IFN-γ (all shown using logarithmic scales). From left to right, are shown results for healthy controls (ANA^-^HC), asymptomatic ANA^+^ individuals (ANA^+^NS), undifferentiated connective tissue disease (UCTD) patients, and systemic autoimmune rheumatic disease (SARD) patients (F, female; M, male). Each circle represents a single subject, with the bars indicating the median for the subjects and error bars denoting the interquartile range. Significant differences between males and females are indicated with asterisks, * p < 0.05, ** p < 0.01, and *** p < 0.001.


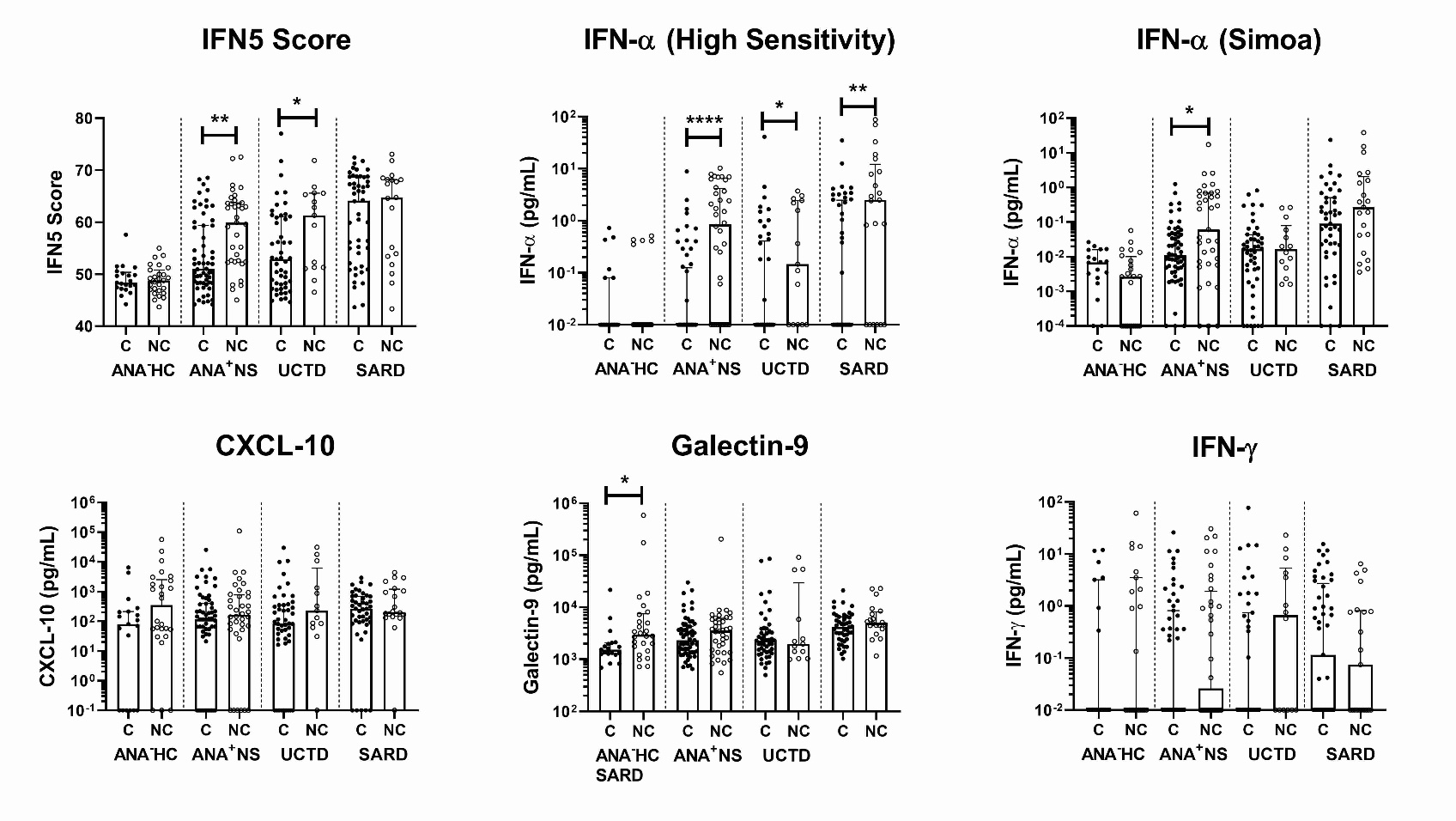


**Supplementary Figure 2.** **Cytokine levels in the ANA^+^ participant subsets stratified by ethnicity.** Scatterplots showing the results for the IFN5 score and the cytokines, IFN-α measured by high sensitivity ELISA, IFN-α measured by Simoa, CXCL-10, Galectin-9, and IFN-γ (all shown using logarithmic scales). From left to right, are shown results for healthy controls (ANA^-^HC), asymptomatic ANA^+^ individuals (ANA^+^NS), undifferentiated connective tissue disease (UCTD) patients, and systemic autoimmune rheumatic disease (SARD) patients (C, Caucasian; NC, non-Caucasian). Each circle represents a single subject, with the bars indicating the median for the subjects and error bars denoting the interquartile range. Significant differences between Caucasians and non-Caucasians are indicated with asterisks, * p < 0.05, ** p < 0.01, and **** p < 0.0001.


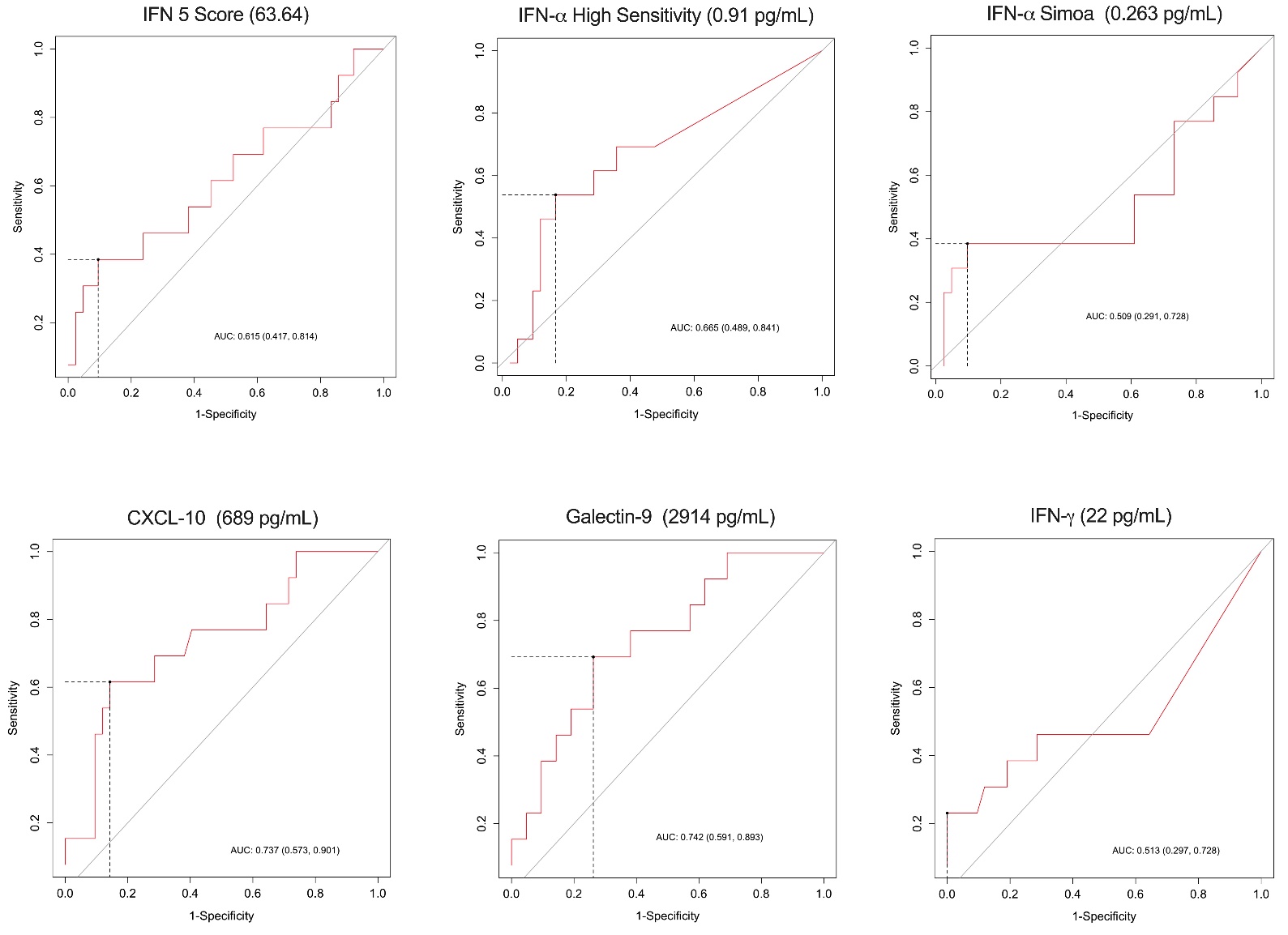


**Supplementary Figure 3. Receiver operating characteristic curves for prediction of clinical progression in subjects followed for ≥ 2 years using the various cytokine measures.** The clinical and serologic characteristics of the clinical progressor (n=13) and non-progressor (n=42) subjects are shown in Table 1. Results are shown for the IFN5 score and the cytokines, IFN-α measured by high sensitivity ELISA, IFN-α measured by Simoa, CXCL-10, Galectin-9, and IFN-γ. Numbers in brackets following the cytokine labels and the black dot on the curve indicate the calculated value of Youden’s Index, comparing progressors and non-progressors. The area under the curve (AUC) is shown in the bottom right corner.
